# Tromethamine improves mucociliary clearance in cystic fibrosis pigs

**DOI:** 10.14814/phy2.15340

**Published:** 2022-09-08

**Authors:** Jamison J. Ash, Brieanna M. Hilkin, Nicholas D. Gansemer, Eric A. Hoffman, Joseph Zabner, David A. Stoltz, Mahmoud H. Abou Alaiwa

**Affiliations:** ^1^ Department of Internal Medicine Pappajohn Biomedical Institute Roy J and Lucille A Carver College of Medicine University of Iowa Iowa City Iowa USA; ^2^ Department of Radiology Roy J and Lucille A Carver College of Medicine University of Iowa Iowa City Iowa USA; ^3^ Roy J Carver, Department of Biomedical Engineering University of Iowa Iowa City Iowa USA; ^4^ Department of Molecular Physiology and Biophysics Roy J and Lucille A Carver College of Medicine University of Iowa Iowa City Iowa USA

**Keywords:** CT imaging, cystic fibrosis, mucociliary transport, therapeutics

## Abstract

In cystic fibrosis (CF), the loss of cystic fibrosis transmembrane conductance regulator (CFTR) mediated Cl^− ^and HCO_3_^−^ secretion across the epithelium acidifies the airway surface liquid (ASL). Acidic ASL alters two key host defense mechanisms: Rapid ASL bacterial killing and mucociliary transport (MCT). Aerosolized tromethamine (Tham) increases ASL pH and restores the ability of ASL to rapidly kill bacteria in CF pigs. In CF pigs, clearance of insufflated microdisks is interrupted due to abnormal mucus causing microdisks to abruptly recoil. Aerosolizing a reducing agent to break disulfide bonds that link mucins improves MCT. Here, we are interested in restoring MCT in CF by aerosolizing Tham, a buffer with a pH of 8.4. Because Tham is hypertonic to serum, we use an acidified formulation as a control. We measure MCT by tracking the caudal movement of individual tantalum microdisks with serial chest computed tomography scans. Alkaline Tham improves microdisk clearance to within the range of that seen in non‐CF pigs. It also partially reverses MCT defects, including reduced microdisk recoil and elapse time until they start moving after methacholine stimulation in CF pig airways. The effect is not due to hypertonicity, as it is not seen with acidified Tham or hypertonic saline. This finding indicates acidic ASL impairs CF MCT and suggests that alkalinization of ASL pH with inhaled Tham may improve CF airway disease.

## Graphical abstract

Cystic fibrosis airways are acidic and as a result, submucosal gland secretions are abnormally elastic. Tromethamine, a tris buffer with long‐lasting effect on pH, alkalinizes the airways and reverses mucociliary clearance defects in CF.

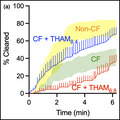

## 1 INTRODUCTION

Cystic fibrosis (CF) is a multi‐organ disorder caused by mutations in the *cystic fibrosis transmembrane conductance regulator (CFTR) gene* and characterized by life‐threatening acute and chronic airway infection, inflammation, and airway remodeling (Welsh et al., 2019, Ratjen et al., 2015, Elborn, 2016, Stoltz et al., 2015). Loss of Cl^−^ and HCO_3_^−^ secretion renders the airway surface liquid (ASL) acidic and this inhibits rapid bacterial killing by antimicrobial peptides and proteins (Poulsen et al., 1994, Pezzulo et al., 2012, Abou Alaiwa et al., 2014, Stoltz et al., 2015). In addition, the acidic environment alters mucus biophysical properties, creating tenacious mucus that is more difficult to transport by the cilia (Birket et al., 2014; Hoegger et al., 2014c; Stoltz et al., 2015).

The airway is a system of branching tubes lined with epithelium which forms a barrier at the interface with the environment (Hsia et al., 2016, Weibel, 1991). In large mammals (including humans), mucus is produced by goblet cells and secretory epithelial cells throughout the airways, and by submucosal glands (SMGs) in large airways (Hovenberg et al., 1996; Okuda et al., 2019; Thornton et al., 2008; Wickstrom et al., 1998). With each breath, inhaled pathogens and particles are trapped by mucus in the airways and swept out of the lungs by the action of cilia (Houtmeyers et al., 1999; Wanner et al., 1996). This process is called mucociliary transport (MCT).

Our recent work highlighted the role of SMG secretions in the mechanism of MCT in large airways (Fischer et al., 2019; Hoegger et al., 2014c; Ostedgaard et al., 2017). Cholinergic stimulation increased mucus secretion from SMGs in the form of strands. These mucus strands facilitated the transport of large particles (Fischer et al., 2019). In CF airways, mucus strands failed to detach from their anchor point at the level of the SMG duct opening (Hoegger et al., 2014c). Instead, they were stretched by the action of cilia and repetitively snapped backward due to abnormal elastic recoil forces (Pino‐Argumedo et al., 2022). Both “failure to detach” and “backward recoil” impaired the movement of particles (Hoegger et al., 2014c; Pino‐Argumedo et al., 2022). Addition of Tris(2‐carboxyethyl)phosphine (TCEP), a reducing agent that severs disulfide bonds, broke down mucus strands, and restored MCT (Pino‐Argumedo et al., 2022).

Mucolytics in clinical use attempt to break up mucus by different mechanisms. DNAses such as Dornase alpha target bacterial DNA in CF mucus (Fuchs et al., 1994; Shak et al., 1990). Osmotic agents such as hypertonic saline (HS) or mannitol alter CF ASL volume (Aitken et al., 2012; Daviskas et al., 1997; King et al., 1997; Robinson et al., 1999). Reducing agents such as N‐acetyl cysteine target disulfide bridges in CF mucus (Nash et al., 2009; Tam et al., 2013). Percussive chest physiotherapy uses mechanical means to mobilize mucus (App et al., 1998; Button & Button, 2013; Sutton et al., 1983). Although some of these agents showed clinical benefit in adults with advanced CF airway disease, it is not clear if they work in children (Amin et al., 2010; Donaldson et al., 2006; Elkins & Bye, 2006; Rosenfeld et al., 2012).

Loss of CFTR‐mediated HCO_3_^−^ secretion acidifies the airways (Poulsen et al., 1994). Inhaled alkaline buffers such as HCO_3_^−^ or glycine are one therapeutic approach to raise pH (Davis et al., 2013; Gomez et al., 2020). Tromethamine (Tham), a tris‐based alkaline buffer with a pKa of 7.82, can alkalinize the airways and unlike HCO_3_^−^ its effects on pH are long‐lasting (Abou Alaiwa et al., 2016). In CF pigs, inhaled alkaline Tham (pH of 8.4) restored the function of antimicrobial peptides and proteins and improved the ability of ASL to quickly kill bacteria (Abou Alaiwa et al., 2016, Holliday et al., 2021). We were motivated to study the effect of alkaline Tham on MCT because of several recent observations suggesting that mucostasis is pathogenic and fixing only the bacterial killing defect may not be sufficient to entirely reverse CF airway disease. Mucus plugging in the βENaC‐Tg and SLC26A9‐KO mice leads to inflammation in the absence of airway infections (Anagnostopoulou et al., 2012; Gehrig et al., 2014; Mall et al., 2004; Zhou et al., 2011). In CF ferrets on lifelong antibiotics, mucus accumulation and inflammation are present despite the eradication of bacterial infection (Rosen et al., 2018). In 3‐week‐old CF pigs on antibiotics since birth, air trapping and mucus plugging persist. Inflammation is present in the absence of detectable bacteria (Bouzek et al., 2021). We wanted to know if correcting the airway pH defect using inhaled alkaline Tham would reverse MCT defects in CF. To control for the hypertonicity of alkaline Tham, we compared Tham to two different interventions: Acidified Tham (pH of 6.8) and hypertonic saline (HS, 7% NaCl, 2400 mOsm/L). The commercially available Tham solution has an osmolarity of 389 mOsm/L (0.3 M).

## 2 METHODS

### 2.1 Animals

Newborn CF pigs were obtained from Exemplar Genetics. We studied male and female pigs 8–15 h after birth. Sedation was with ketamine (20 mg/kg, i.m., Phoenix Pharmaceutical, Inc.) and acepromazine (2 mg/kg, i.m., Phoenix Pharmaceutical, Inc.) or xylazine (2 mg/kg, i.m., Lloyd) and anesthesia was maintained with i.v. dexmedetomidine (10 µg/kg/h, i.v., Accord Healthcare, Inc.). Euthanasia was with i.v. Euthasol (Virbac).

### 2.2 Delivery of agents into the airways

We measured MCT *in vivo* before and after stimulating submucosal gland secretion. We administered the cholinergic agonist methacholine (Methapharm) intravenously (i.v.) as previously reported (Hoegger et al., 2014c; Ostedgaard et al., 2017; Pezzulo et al., 2012). As a control for hypertonicity, we used acidified Tham (Pfizer Hospital), titrated with glacial acetic acid (Fischer Scientific) to a pH of 6.8. We chose a pH of 6.8 because the optimal buffering range of tris buffer is between 6.8 and 9 (Kaplan, 1962, Kresh et al., 1987). We aerosolized each intervention with a volume of 0.5 ml using an atomizer. The expected droplet size is in the range of 30–100 µm with projected deposition in the large airways including the trachea (Heyder, 2004, Asgharian et al., 2016). In some experiments we aerosolized hypertonic saline (7%, RPI, Mt Prospect), acidified Tham (pH 6.8), or alkaline Tham (pH 8.4) concurrently with methacholine and acquired CT scans (HS, *N* = 5; Tham pH 6.8, *N* = 6; Tham pH 8.4, *N* = 7). All aerosolized interventions were done using a MADgic microsprayer (Teleflex).

### 2.3 *In vivo* MCT assay

To measure MCT in vivo, we used a previously described X‐ray computed tomographic (CT) assay (Fischer et al., 2019; Hoegger et al., 2014a, 2014b,2014a, 2014b; Pino‐Argumedo et al., 2022). We measured MCT by tracking tantalum microdisks (350 μm diameter × 25 μm thick, Millipore Sigma). To deliver microdisks, animals were anesthetized, briefly intubated, and microdisks were insufflated into the airways just beyond the vocal cords with a puff of air. Immediately after delivery, the tube was removed. CT scans were acquired with a dual source high‐resolution multi‐row detector CT scanner (Siemens SOMATOM Force, Siemens Healthineers). 44 CT scans were obtained in a 6.3 min time interval. Microdisks were tracked over time as previously described (Hoegger et al., 2014a). Tracking microdisks over time provided multiple instantaneous measurements of microdisk speed. From these speeds, we determined individual microdisk maximum speed and mean speed for microdisks that moved >10 mm. Microdisk clearance was determined by measuring whether a microdisk left the tracking field or not during the 6.3 min tracking period. The percentage of microdisks cleared was determined by dividing the number of cleared microdisks by the total number of microdisks tracked x100%. The data were obtained by a single external observer blinded to treatment conditions.

### 2.4 Freeze‐fracture scanning electron microscopy

Trachea samples were removed from newborn CF pigs at the time of necropsy, flash frozen in liquid nitrogen and maintained at the temperature of liquid nitrogen throughout the procedure. The Quorum cryo‐stage system (Electron Microscopy Sciences) was used on the Hitachi SU‐4800 field emission SEM (Hitachi High Technologies America, Inc). Tracheal portions were attached to the cryo‐holder using a cryo‐carbon compound, and immediately frozen in liquid nitrogen slurry using the Quorum plunge freeze system to fix the tissue to the holder. The frozen sample was quickly transferred to the prep station and pumped to a high vacuum while kept under cold temperatures. In the prep station, the sample was fractured in a plane perpendicular to the lumen central axis and sublimated at −90°C for 10 min and then lightly sputter coated with gold (30 s) to create a conductive surface. The coated frozen sample was then transferred into the SEM chamber cryo‐stage for imaging at 10 kV. The holder, prep stage and cryo‐stage were all maintained at −140 C.

### 2.5 Statistical analysis

Differences were considered statistically significant at *p* < 0.05. All analyses were completed in GraphPad Prism v9.3.0 (GraphPad Software). Data from individual animals are presented as individual data points and mean ± SEM are indicated by bars. For comparisons, we used Mann–Whitney test.

## 3 Study approval

The present studies in animals were reviewed and approved by the University of Iowa Animal Care and Use Committee.

## 4 RESULTS

### 4.1 Alkaline Tham increased clearance in CF pigs after cholinergic stimulation

**FIGURE 1:**
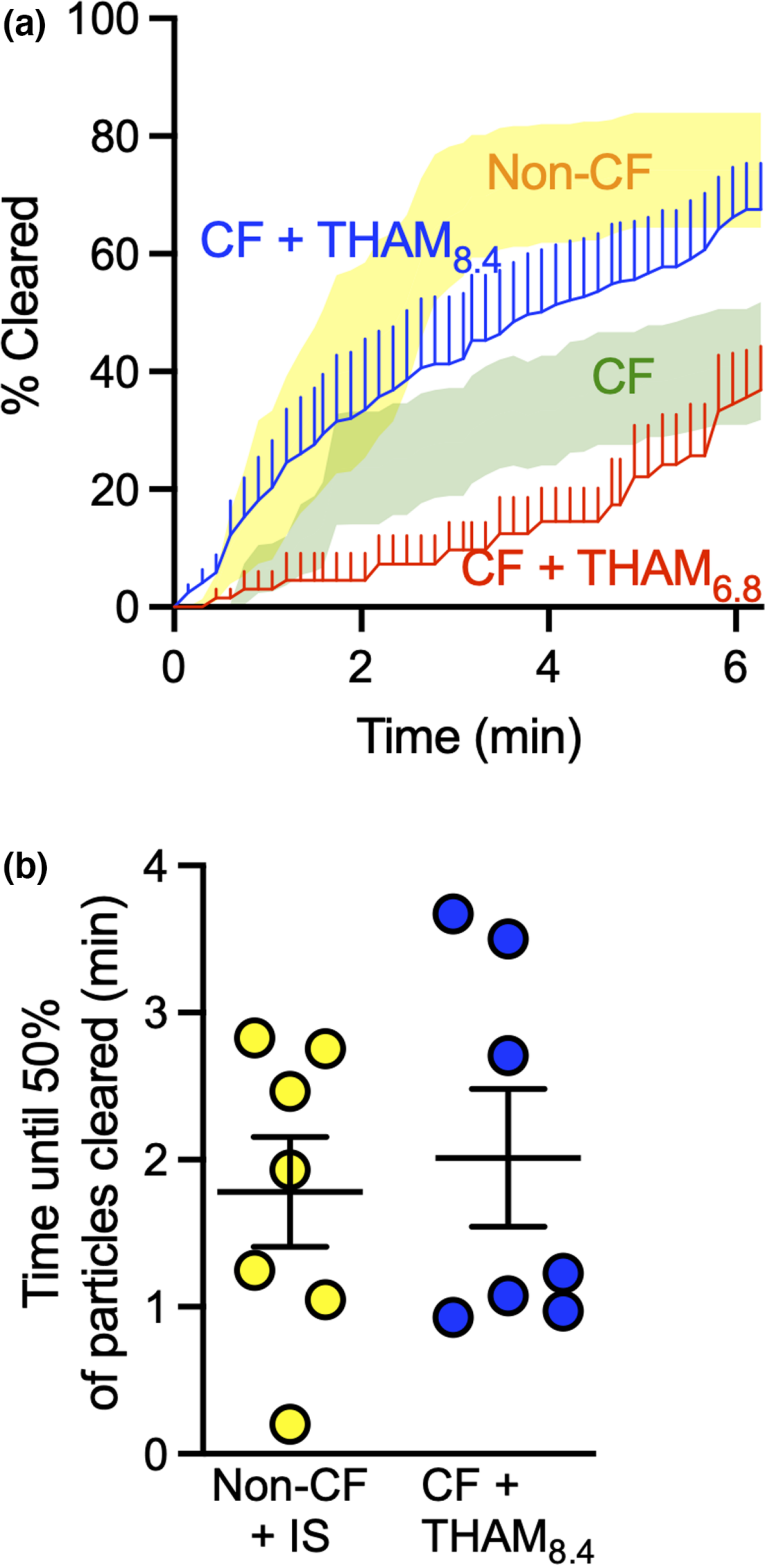
Aerosolized alkaline Tham increases microdisks clearance from CF airways. (a) Percent of microdisks cleared from the field. Lines represent mean and standard error for acidified Tham at pH 6.8 (red) and alkaline Tham at pH 8.4 (blue). 95% confidence interval bands reproduced from earlier publication and included here for comparison (yellow band represent vehicle‐treated non‐CF from Ref. 18, green band represent vehicle‐treated CF from Ref. 20). (b) Time until half the microdisks cleared the airways. Lines and error bars represent mean ± SEM, *N* = 6–7 different pigs.

To measure MCT in CF pigs, we tracked the position and movement of individual tantalum microdisks and calculated the percentage that cleared the lungs as previously described (Hoegger et al., 2014a,b,c, Fischer et al., 2019, Pino‐Argumedo et al., 2022). Our earlier finding that Tham raises ASL pH *in vivo* with a long‐lasting effect and reverses bacterial killing defects in CF airways encouraged us to test if it also improves MCT (Abou Alaiwa et al., 2016, Holliday et al., 2021). Microdisk clearance with alkaline Tham averaged 68 ± 8% similar to what we reported before in normal airways with a mean of 74% [95% confidence interval: 50, 98] (yellow band) (Figure 1a) (Fischer et al., 2019). In contrast, with acidic Tham (pH 6.8) microdisk clearance averaged 37 ± 7% in the range of what we reported before in CF airways treated with isotonic saline with a mean of 42% [95% confidence interval: 20, 63] (green band) (Pino‐Argumedo et al., 2022).

In non‐CF airways, microdisks clearance seemed to plateau faster than in CF pigs treated with alkaline Tham (Figure 1a) (Fischer et al., 2019). However, when we calculated the average time it took for 50% of the microdisks to clear, there was no statistically significant difference (1.8 ± 0.4 min vs. 2.0 ± 0.5 min) (Figure 1b). These data suggest that alkaline Tham can restore MCT of microdisks in CF large airways.

These results are comparable to previous data with the reducing agent TCEP in CF. With inhaled TCEP, microdisk clearance averaged 61 ± 10% (Figure S1) (Pino‐Argumedo et al., 2022).

### 4.2 Alkaline Tham partially restores two MCT defects in CF pigs after cholinergic stimulation

**FIGURE 2:**
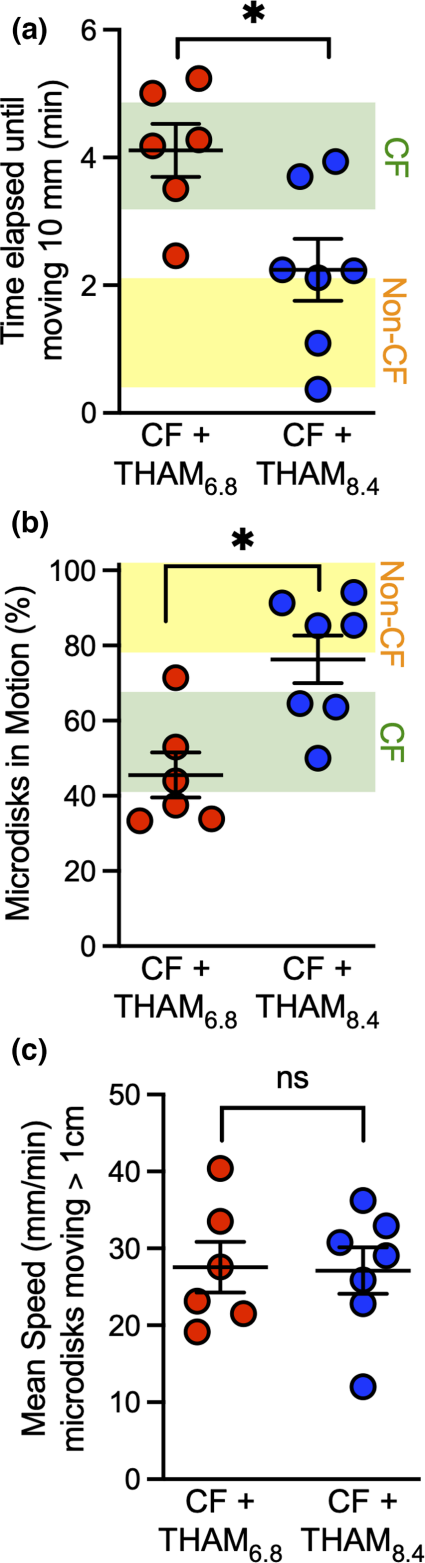
Aerosolized alkaline Tham increases microdisk movement. (a) Elapsed time until microdisks moved 10 mm. (b) % microdisks in motion. (c) Average speed of moving microdisks. Each set of data points is from a different pig. Some data points overlap. Lines and error bars represent mean ± SEM. Ninety five percent confidence interval bands reproduced from earlier publication and included here for comparison (yellow band represent vehicle‐treated non‐CF from Ref. 18, green band represent vehicle‐treated CF from Ref. 20). * *p* < 0.05 by Mann–Whitney test, *N* = 6–7 different pigs.

Tham_8.4_ decreased the time elapsed until microdisks moved at least 10 mm from their starting position to 2.2 ± 0.5 min in comparison to 4.1 ± 0.4 min with Tham_6.8_. Because of respiratory the oscillations we used 10 mm as a cutoff to determine microdisks movement. These values are very close to the range we previously reported in non‐CF airways treated with isotonic saline with a mean of 1.1 min [95% confidence interval^:^ 0.14, 2.11] (Fischer et al., 2019) (Figure 2a). In addition, over the 6.3 min period of observation, Tham_8.4_ increased the percentage of microdisks in motion to 76 ± 6% in comparison to acidic Tham 46 ± 6%. In earlier reports, we found that in CF pig airways the majority of microdisks failed to move (Hoegger et al., 2014c; Pino‐Argumedo et al., 2022). These values are in the range of non‐CF airways treated with isotonic saline with a mean of 90% [95% confidence interval: 78, 102] (Figure 2b) (Fischer et al., 2019). Interestingly, there was no difference in the mean speed of moving particles between alkaline Tham and acidic Tham (Figure 2c). Although the average speed of these microdisks is high in comparison to what is reported in the literature (Cooper et al., 2013; Liu et al., 2013), the values are comparable to the increased microdisk speed that we have seen with cholinergic stimulation (Hoegger et al., 2014a,b,c, Fischer et al., 2019, Pino‐Argumedo et al., 2022).

### 4.3 Alkaline Tham decreased the number of backward recoils

**FIGURE 3:**
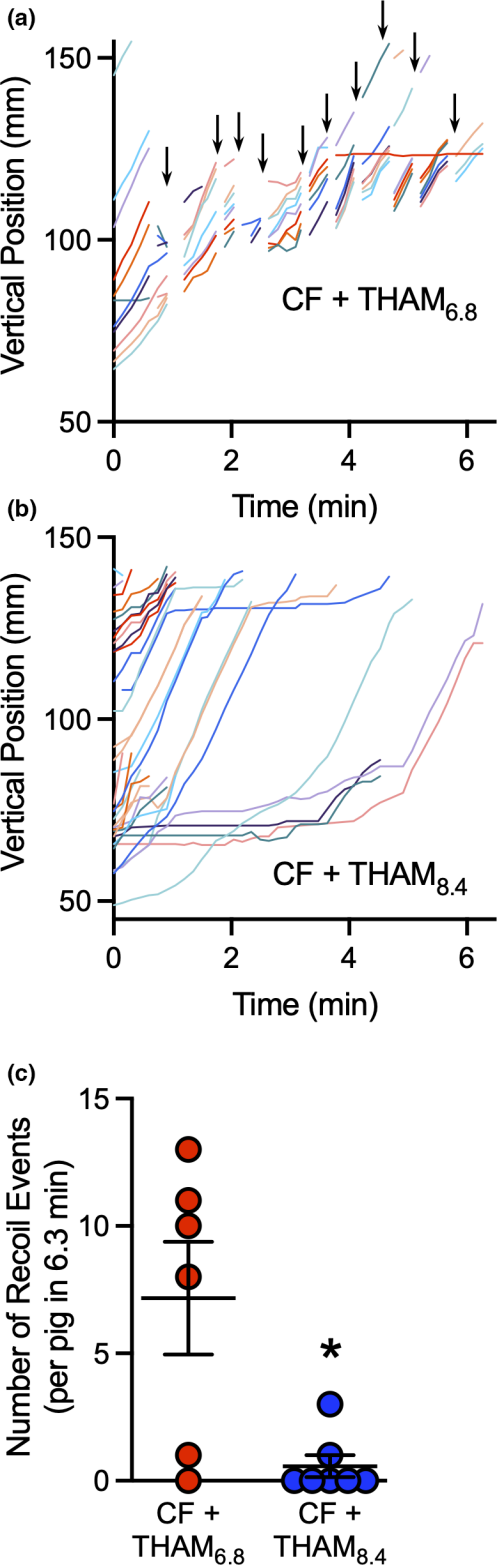
Aerosolized alkaline Tham largely eliminates recoil transport of microdisks. (a & b) Data show vertical positions of microdisks as they are transported in two representative experiments in CF pigs. Each microdisk is given a unique color. After simultaneous treatment with IV methacholine and inhaled acidified or alkaline Tham, microdisk moved at the same speed (slope of line segments). (a) Backward recoil with acidified Tham. Discontinuous traces indicate times when microdisks abruptly moved backwards (see video). (b) With alkaline Tham, microdisks advanced through the airway without apparent recoil events. (c) Number of recoil events per animal. Lines represent mean and standard error for inhaled acidifed Tham (red) or alkaline Tham (blue). *p < 0.05 by Mann–Whitney test, *N* = 6–7 different pigs.

Our earlier observation of abrupt retrograde microdisk movement after inhaled saline and cholinergic stimulation is reproduced here with Tham_6.8_ and cholinergic stimulation. MCT is one‐directional with mucus always transporting towards the larynx. We described a phenomenon in CF pigs that manifested after inhaled isotonic saline and i.v. methacholine whereby the forward transport of microdisks was repeatedly and abruptly interrupted with a sudden backward recoil. This abrupt recoil was much faster than our image acquisition leading to interrupted and discontinuous tracking. The elastic recoil of mucus strands pulled the microdisks fast, we could not with certainty identify the same microdisk after each recoil. Instead, we quantified the number of discontinuities in each trajectory. Similar to inhaled saline concomitantly with i.v. methacholine, Tham_6.8_ with i.v. methacholine induced substantial backward recoil of 7.2 ± 2.2 events per pig during the 6.3 min of the study (Figure 3a and c, Video S1). In contrast, there were far fewer recoil events demonstrated in pigs treated with Tham_8.4_ (0.7 ± 0.4) (Figure 3b and c, Video S2).

### 4.4 HS does not restore MCT defects in CF pigs after cholinergic stimulation

**FIGURE 4:**
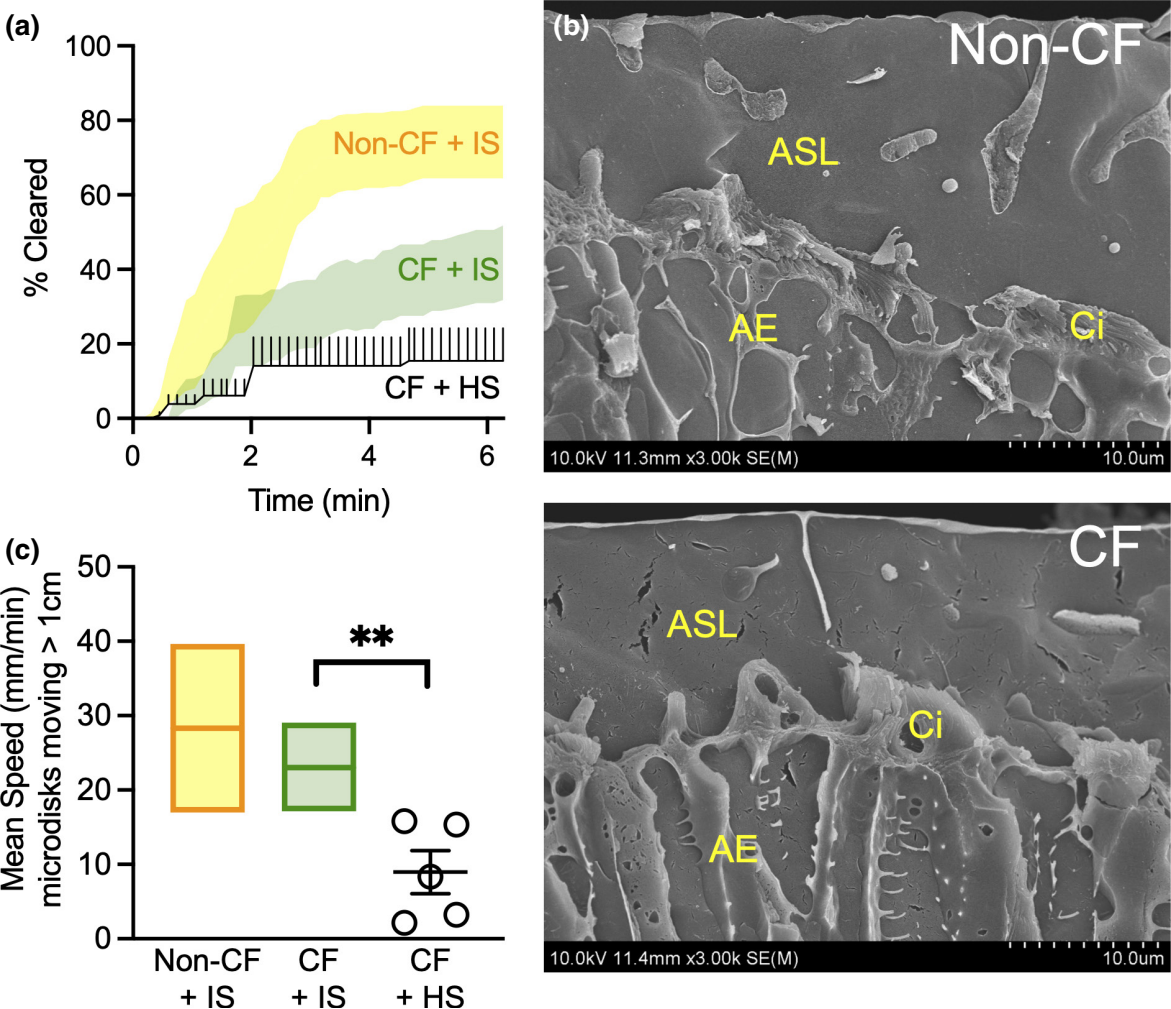
Aerosolized HS decreases microdisk movement in CF pigs. (a) Percent of microdisks cleared from the field. Lines represent mean and standard error for HS (black). 95% confidence interval bands reproduced from earlier publication and included here for comparison (yellow band represent vehicle‐treated non‐CF from Ref. 18, green band represent vehicle‐treated CF from Ref. 20). (b) Scanning electron micrograph of a freeze‐fracture preparation of non‐CF and CF pig trachea after cholinergic stimulation. The fracture plane was passed orthogonal to the luminal surface of the epithelium. (ASL) Airway Liquid Surface. (Ci) Cilia. (AE) Airway Epithelium. Scale bar =10 µm. (c) Average speed of moving microdisks. Each set of data points is from a different pig. Lines and error bars represent mean ± SEM. Mean and 95% confidence interval bands reproduced from earlier publication and included here for comparison (yellow band represent vehicle‐treated non‐CF from Ref. 18, green band represent vehicle‐treated CF from Ref. 20). **p < 0.01 by Mann–Whitney test, *N* = 5 different pigs.

In CF pigs, host defense defects became apparent after stimulation of SMG secretion with i.v. methacholine (Hoegger et al., 2014c; Ostedgaard et al., 2017; Pezzulo et al., 2012). We asked if MCT changed after aerosolizing HS at the same time as we stimulate SMG secretion in CF pigs. After 6.3 min, microdisk clearance averaged 18 ± 7% (Figure 4a). These values were lower than what we reported previously with inhaled isotonic saline in non‐CF airways with mean of 74% [95% Confidence Interval: 50, 98] (yellow band) and lower than with inhaled isotonic saline in CF airways with mean of 42% [confidence interval: 20, 63] (green band) (Figure 4a) (Fischer et al., 2019, Pino‐Argumedo et al., 2022).

HS can affect MCT by increasing ASL volume due to its osmotic effect (Goralski et al., 2018). Cholinergic stimulation increases SMGs liquid secretion in bovine and in both CF and non‐CF porcine trachea (Lee & Foskett, 2014, Widdicombe, 2002, Joo et al., 2010). We did not pursue a quantification of the effect of HS on ASL height, as within minutes of methacholine administration both CF and non‐CF airways are already flooded (Figure 4b).

Our analysis of individual microdisks in the airways revealed why HS failed to increase clearance. The mean speed of moving microdisks decreased with HS to 9 ± 3 mm/min in comparison to isotonic saline in CF airways with mean of 23 mm/min [95% confidence interval: 17, 29] or non‐CF airways with mean of 28 mm/min [95% confidence interval: 17, 40] (Figure 4c) (Hoegger et al., 2014a,b,c, Pino‐Argumedo et al., 2022). The data are consistent with slowed transport of particles, and this may be explained by a decrease in CBF with HS (Boek et al., 1999; Kelly et al., 2021; Min et al., 2001). Perhaps because of slower speed of transport, we did not observe any abrupt backward recoil events in pigs treated with HS.

## 5 DISCUSSION

Our data indicate that increasing ASL pH with inhaled buffers reverses microdisk MCT defects in large CF airways. The effect is not solely due to increased tonicity of Tham because an acidified Tham solution of similar tonicity did not restore MCT.

Alkaline Tham increased microdisk clearance in CF airways. However, acidified Tham had no effect. These results suggest that alkalinizing ASL pH may be beneficial in CF. Analyzing the trajectories of individual microdisks points to the underlying mechanism. We note that under baseline conditions there were no recoils and with alkaline Tham the frequency of backward recoils decreased. This increased clearance is comparable to our published data that MCT was improved with the addition of reducing agents (Pino‐Argumedo et al., 2022) and suggests increasing ASL pH alters mucus biophysical properties (Tang et al., 2016).

These findings were surprising because our recent data show that once mucus has expanded and formed in an abnormal environment, it becomes resistant to further changes. First, adding HEPES buffer at a pH of 7.4 did not correct mucus properties and SMG mucus strands failed to detach (Hoegger et al., 2014c). Second, banana slug granule mucus formed at acidic pH demonstrated abnormal viscoelastic properties. Raising the pH to very alkaline values (pH 9) did not reverse these properties (Xie et al., 2020). Based on these data suggesting mucus is non‐modifiable when formed in an abnormal environment, we speculate that alkaline Tham may be modifying the properties of nascent mucus strands. This freshly secreted mucus now enters an ASL environment that is favorable to forming mucus correctly and therefore the biophysical properties would allow the mucus to break. This is similar to another study demonstrating that the addition of apical bicarbonate corrected CF mouse gut mucus detachment abnormalities (Gustafsson et al., 2012).

A surprising finding was that inhaled HS did not improve MCT in CF airways. We found that the addition of HS resulted in decreased mean speed of particles without impacting the percentage of microdisks in motion or the time they start to move. This decrease in mean speed subsequently resulted in decreased particle clearance. At first, this result seems in conflict with the current consensus on HS. HS is a component of CF treatment because it is believed to improve MCT, improve CF symptoms and improve short‐term outcomes (Donaldson et al., 2006; Elkins et al., 2006). However, this discrepancy of decreased MCT may be explained by understanding how HS is affecting the airway epithelium within our experimental context. First, HS is an osmotic mucolytic and as such it may draw liquid into ASL and improve MCT by increasing ASL height (Goralski et al., 2018; Luan et al., 2019). However, in the presence of cholinergic stimulation, fluid secretion is already maximally stimulated (Lee & Foskett, 2014). We did not measure ASL height in pigs given inhaled HS and stimulated with i.v. methacholine because it is possible that the ASL depth would be greater than with methacholine alone. In a flooded trachea, excessive increase in ASL depth may inhibit MCT. However, in submerged preparations of airways, cilia was still able to generate the transport of mucus (Fischer et al., 2019; Hoegger et al., 2014c; Pino‐Argumedo et al., 2022). Second, HS is an irritant and may induce MCT by provoking cough reflexes (Robinson et al., 1996). The cough mechanism was suppressed in our study from deep sedation. Third, the beneficial effect of HS may depend on the status of the airways and if there is already bronchiectasis and remodeling. This may explain why the clinical benefits of HS are seen only in adults with advanced CF airway disease (Donaldson et al., 2006; Elkins & Bye, 2006; Elkins et al., 2006). However, in young children with CF, there is no improvement with HS despite some changes in lung clearance index (Amin et al., 2010; Laube et al., 2011; Rosenfeld et al., 2012). Fourth, HS may be slowing cilia and subsequently lowering mucus transport speed in the airways. Prior studies reported ciliostasis with 7% HS (Boek et al., 1999; Kelly et al., 2021; Min et al., 2001). Thus, in our experiment of newborn pigs, without progressive disease and remodeling, we may be seeing the impact of HS acting largely on the ciliary component of MCT. This decreased CBF in conjunction with abnormal mucus biophysical properties resulted in clearance worse than CF pigs treated with IS.

Highly effective CFTR modulators targeting CF mutations are currently available, with others in the pipeline. However, there are many CF mutations that are not amenable to the effects of these drugs (Lopes‐Pacheco, 2019). In addition, a number of people with CF do not tolerate the adverse effects of CFTR modulators (Burgel et al., 2020; Siracusa et al., 2015). Inhaled buffers may provide the benefit of both improving the ability of ASL to rapidly kill microbes and correcting the biophysical properties of mucus in these individuals not able to use these new medications. CFTR modulators should change CFTR function everywhere, including within SMG’s acini and ducts, while inhaled buffer effect is localized to the surface epithelium. But this may be sufficient to restore normal airway function and inhaled buffers could likely be used in combination with CFTR modulators for additive benefits.

### 5.1 Advantages and limitations

This study has many advantages. First, the airways of pigs serve as an excellent model for human because they have SMGs and develop the hallmarks of the human CF airway disease (Choi et al., 2000; Hajighasemi‐Ossareh et al., 2013). Second, we study newborn CF pigs, at a key age where the lungs are clear of infection and inflammation, yet they manifest CF host defense defects (Stoltz et al., 2015). Third, we study inhaled buffer with long‐lasting effect on pH (Abou Alaiwa et al., 2016). Because the protonated form of Tham may penetrate the cells slowly, it may linger in the airways (Brasch et al., 1982). Fourth, Tham will shift the CO_2_−HCO_3_^−^ balance towards generating HCO_3_^−^ (Nahas et al., 1998). Fifth, Tham is FDA approved for intravenous use (Brasch et al., 1982; Kresh et al., 1987). It has a favorable toxicity profile and is already used as an excipient for many preparations including nasal Ketorolac and inhaled Iloprost (Quadir et al., 2000).

This study is limited to transport of large particles in large airways. Neither our CT scan assay nor our intervention targets the small airways (Fischer et al., 2019; Hoegger, Awadalla, Namati, et al., 2014; Hoegger et al., 2014c; Pino‐Argumedo et al., 2022). The microdisks are large and deposit in the large airways. We delivered our intervention with an atomizer with large droplet size predicted to deposit in the large airways. Although it is commonly stated that CF airway disease starts in the small airways, direct evidence of this implication is missing because methods to study small airways are lacking.

This work together with our prior studies repurposes Tham for inhaled therapeutic use in CF airway disease. In addition to its long‐lasting effect on ASL pH, Tham enhanced the ability of ASL to rapidly kill bacteria and reversed MCT defects in CF airways. As an inhaled intervention, Tham may find additional use in other airway diseases where abnormalities in mucus and mucus transport are involved such as chronic obstructive pulmonary disease, primary ciliary dyskinesia, asthma, or idiopathic pulmonary fibrosis.

## AUTHOR CONTRIBUTIONS

DAS, JZ and MHAA conceived and designed studies. JJA, BMH, NDG, and MHAA conducted experiments and acquired data. JJA, EAH, DAS, JZ, and MHAA analyzed data. JJA, DAS, JZ and MHAA wrote the manuscript.

## 6 CONFLICT OF INTEREST

The author(s) declared the following potential conflicts of interest with respect to the research, authorship, and/or publication of this article. The University of Iowa Research Foundation has licensed intellectual property related to gene modified pigs to Exemplar Genetics. Royalties from that license are shared with DAS. DAS has other financial ties to Exemplar Genetics. EAH is a founder and shareholder of VIDA Diagnostics, a company commercializing lung image analysis software developed, in part, at the University of Iowa. JZ, DAS and MHAA have a patent application pending related to this work. The remaining authors declare no competing interest.

## Supporting information




Video S1:

Video S2:

Figure S1:

## Data Availability

All study data are included in the article and/or supporting information.
