# Supplementary figures and images for: Tromethamine improves mucociliary clearance in cystic fibrosis pigs
Source: Physiol Rep. 2022 Sep 8;10(17):e15340. doi: 10.14814/phy2.15340 (PMC9453173; doi:10.14814/phy2.15340)

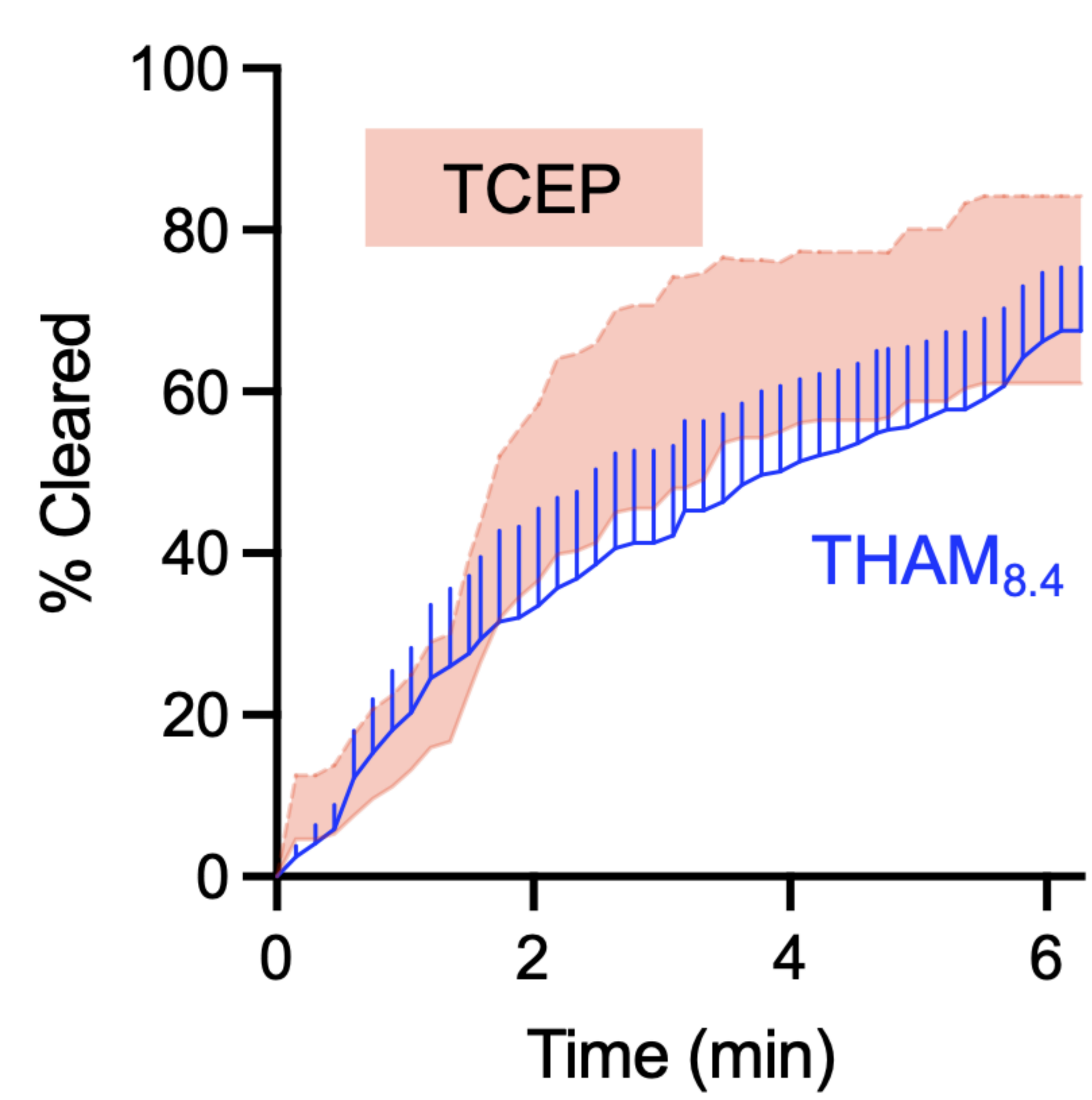

Suppl. Figure

Supplement: Supplementary file 3 — Figure S1: [file PHY2-10-e15340-s002.pdf]
